# Robot-assisted thoracoscopic resection of a costal cartilage chondrosarcoma mimicking anterior mediastinal teratoma: a case report

**DOI:** 10.1186/s44215-026-00279-w

**Published:** 2026-10-01

**Authors:** Takumi Takemoto, Junichi Murakami, Sota Yoshimine, Toshiki Tanaka, Akihito Mikamo

**Affiliations:** Department of Surgery and Clinical Science, Yamaguchi University School of Medicine, 1-1-1 Minami-Kogushi, Ube, Yamaguchi, 755-8505 Japan

**Keywords:** Chondrosarcoma, Costal cartilage, Mature teratoma, Anterior mediastinal tumor, Robot-assisted thoracoscopic surgery

## Abstract

**Background:**

Chondrosarcoma arising from the costal cartilage is extremely rare. When such a tumor extends toward the mediastinum, it may present as a well-defined mass that is difficult to distinguish from a primary anterior mediastinal tumor. We report a case diagnosed preoperatively as a mature teratoma but later proven to be a grade 1 chondrosarcoma of the costal cartilage.

**Case presentation:**

A 66-year-old asymptomatic woman was incidentally found to have an abnormal shadow on chest imaging. Contrast-enhanced computed tomography revealed a well-defined, calcified mass measuring 37 × 31 × 21 mm in the anterior mediastinum, adjacent to the left costal arch; bone-window and reformatted images did not clearly demonstrate continuity with the adjacent costal cartilage. Magnetic resonance imaging demonstrated T1-weighted hyperintensity with signal loss on fat-suppressed imaging, consistent with a fatty component, and no invasion of surrounding structures. Positron emission tomography-computed tomography showed low fluorodeoxyglucose uptake and no distant metastasis, and tumor markers were normal. A preoperative diagnosis of mature teratoma was made, and resection was planned. Robot-assisted thoracoscopic surgery was performed via a three-port right-sided approach. Intraoperatively, the tumor lay in the narrow space between the heart and the sternum, with direct continuity with the left sixth costal cartilage suggesting its origin; the tumor-cartilage boundary could not be identified, and the cartilage was divided at the tumor interface. The postoperative course was uneventful. Pathological examination revealed chondrocyte-like tumor cells with mild nuclear atypia, consistent with a grade 1 chondrosarcoma of the costal cartilage; residual tumor could not be excluded (Rx). A multidisciplinary tumor board elected surveillance, and no recurrence has been observed at 18 months postoperatively.

**Conclusions:**

Costal cartilage chondrosarcoma belongs in the differential diagnosis of a calcified anterior mediastinal mass because preoperative recognition determines whether a wide or marginal resection is planned, and a minimally invasive approach must not compromise the surgical margin when malignancy is suspected. The right-sided robot-assisted thoracoscopic approach provided favorable visualization and maneuverability in this confined retrosternal location, but its oncological safety cannot be generalized from a single case.

## Background

Chondrosarcoma is the most common primary malignant tumor of the chest wall, with an annual incidence below 0.5 per million [1]. Origin from the costal cartilage is rarer still, and when such a tumor extends toward the mediastinum it may mimic a primary anterior mediastinal tumor such as thymoma or mature teratoma, making preoperative differentiation on imaging alone difficult. A previous review found eight reported anterior mediastinal chondrosarcomas, only three arising from the sternum or costal cartilage [2]. With that report and subsequent cases, five chest wall-derived cases have been described, summarized with the present case in Table 1 [2–6].

**Table 1:** Reported cases of chest wall-derived chondrosarcoma presenting as an anterior mediastinal mass

Author (year)	Age/Sex	Origin	Size (cm)	Preoperative diagnosis	Surgical approach	Margin	Grade	Outcome
Phillips and Choong (1991) [5]	NR	Costal cartilage (presumed)	NR	Anterior mediastinal mass	NR	NR	NR	NR
Alpert et al. (2011) [3]	NR	Sternum (periosteal)	NR	Teratoma among differentials	Resection	NR	Periosteal	NR
Nosotti et al. (2012) [4]	71/F	Sternum (posterior cortex)	10 × 7	Thymoma	Sternotomy; subtotal sternectomy with costal cartilages, allograft reconstruction	En bloc	NR	No relapse at 12 months
Østergaard et al. (2015) [2]	75/M	Costal cartilage	4.3 × 2.6 × 3.3	Thymoma	Right VATS excision, then second operation with wider chest wall resection	Re-resection	1	No tumor on MRI at 6 months
Yang et al. (2025) [6]	32/F	Right 5th costal cartilage (HME)	4.7 × 4.0 × 2.3	Anterior mediastinal tumor	Subxiphoid thoracoscopic, intralesional	Intralesional	Low	No recurrence at 12 months (IMRT)
Present case	66/F	Left 6th costal cartilage	3.7 × 3.1 × 2.1	Mature teratoma	Right-sided RATS; cartilage divided at tumor interface	Rx	1	No recurrence at 18 months

*HME* hereditary multiple exostoses, *IMRT* intensity-modulated radiotherapy, *MRI* magnetic resonance imaging, *NR* not reported, *RATS* robot-assisted thoracoscopic surgery, *Rx* margin status cannot be assessed, *VATS* video-assisted thoracoscopic surgery

Wide resection with adequate margins is the standard treatment. Tumors confined to the narrow retrosternal space, however, are technically demanding to reach by median sternotomy or conventional video-assisted thoracoscopic surgery (VATS), and robot-assisted thoracoscopic surgery (RATS) has been applied in such anatomically restricted corridors.

Herein we report a costal cartilage chondrosarcoma continuous with the sixth costal cartilage in which, unlike previously reported cases, mature teratoma was the confident preoperative diagnosis, resected using a three-port right-sided RATS approach.

## Case presentation

**Fig. 1:**
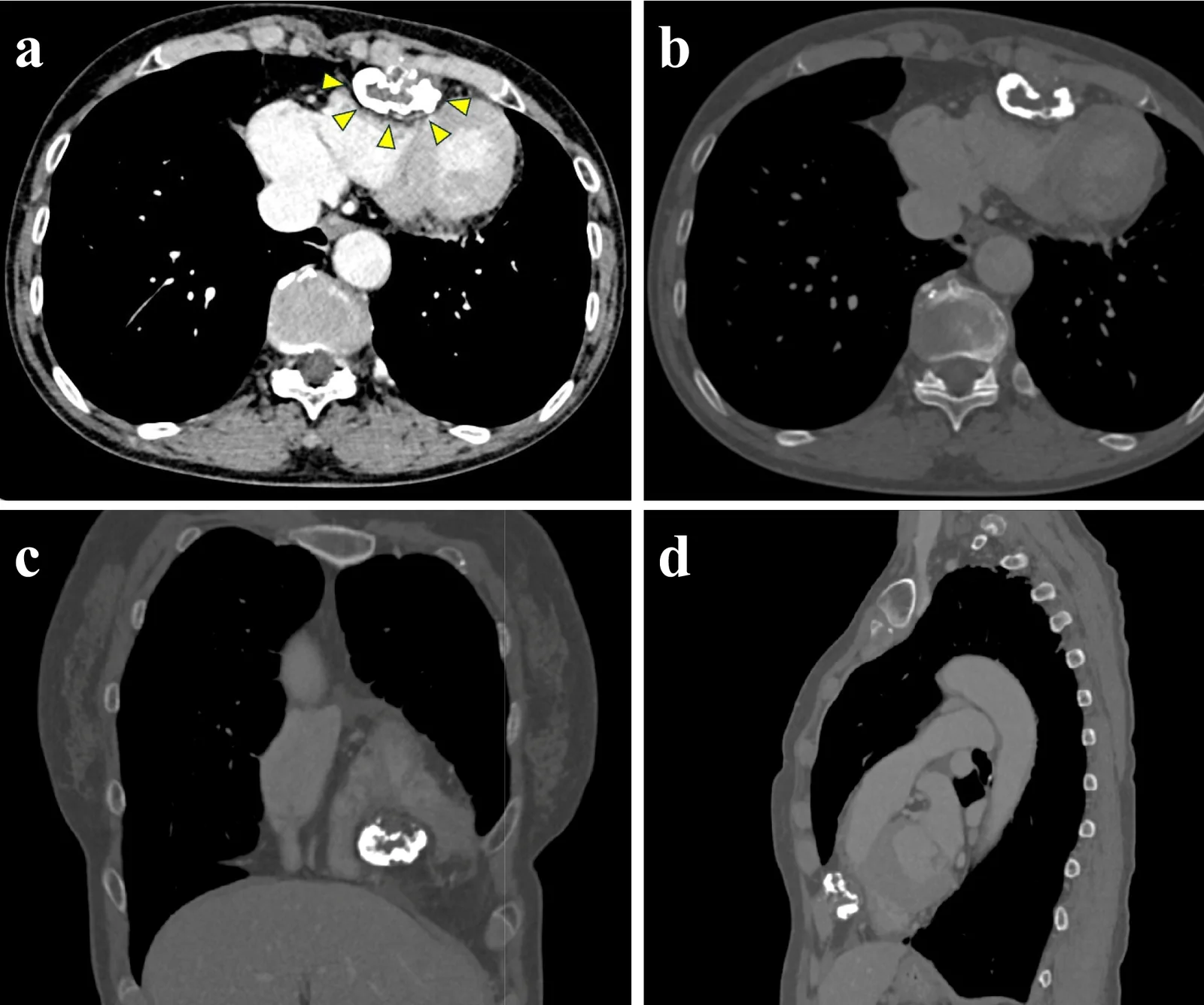
Preoperative computed tomography findings. **a** Contrast-enhanced computed tomography reveals a well-defined, calcified mass measuring 37 × 31 × 21 mm, located adjacent to the left costal arch near the epigastric region. **b** Bone-window computed tomography does not clearly demonstrate continuity between the mass and the adjacent costal cartilage. c Coronal and d sagittal reformatted bone-window images similarly do not delineate the relationship between the tumor and the costal cartilage

**Fig. 2:**
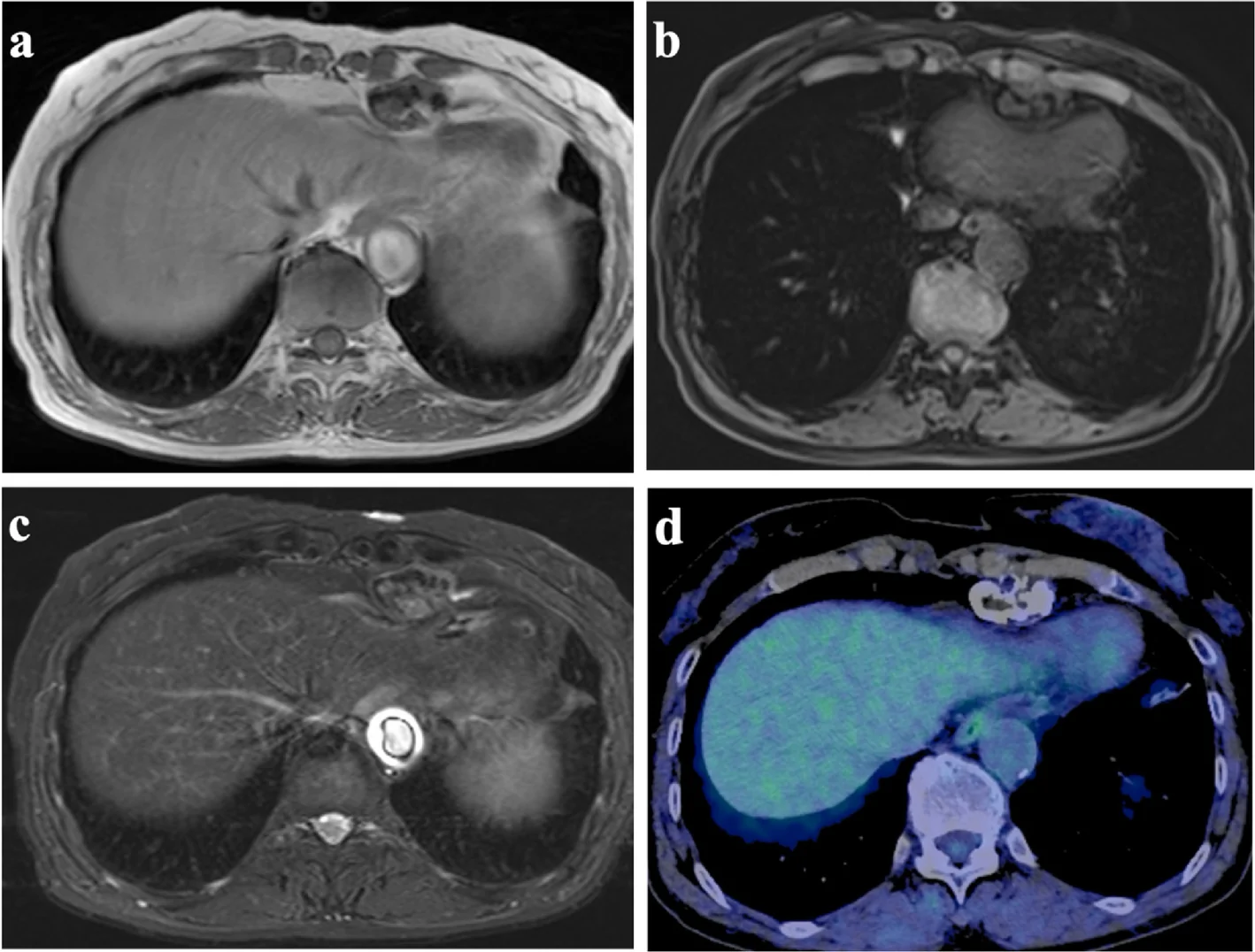
Preoperative magnetic resonance imaging and positron emission tomography-computed tomography findings. **a** T1-weighted imaging demonstrates hyperintensity within the tumor, suggestive of a fatty component. **b** Fat-suppressed T1-weighted imaging shows signal loss in the corresponding area, supporting the presence of fat. **c** T2-weighted imaging shows low signal intensity within the calcified portion of the tumor and high signal intensity in the remaining internal areas. **d** Positron emission tomography-computed tomography shows low fluorodeoxyglucose uptake within the mass

A 66-year-old asymptomatic woman was incidentally found to have an abnormal shadow on chest imaging, with no relevant medical history or carcinogen exposure. Contrast-enhanced computed tomography (CT) revealed a well-defined, poorly enhancing, calcified mass measuring 37 × 31 × 21 mm in the anterior mediastinum, adjacent to the left costal arch near the epigastric region (Fig. 1a). Bone-window CT, together with coronal and sagittal reformats, did not clearly demonstrate continuity between the mass and the adjacent costal cartilage (Fig. 1b-d). Magnetic resonance imaging (MRI) showed T1-weighted hyperintensity with signal loss on fat-suppressed imaging, consistent with fat (Fig. 2a, b); T2-weighted imaging showed low signal within the calcified portion and high signal elsewhere, with no invasion of surrounding structures (Fig. 2c). Positron emission tomography-computed tomography (PET-CT) showed low fluorodeoxyglucose (FDG) uptake and no distant metastasis (Fig. 2d). Tumor markers were within normal limits: carcinoembryonic antigen (CEA) 2.8 ng/mL (reference range < 6.0 ng/mL), alpha-fetoprotein (AFP) 4.3 ng/mL (reference range < 10.0 ng/mL), and squamous cell carcinoma antigen (SCC) 0.5 ng/mL (reference range < 1.5 ng/mL). A preoperative diagnosis of mature teratoma was made, and resection was planned.

**Fig. 3:**
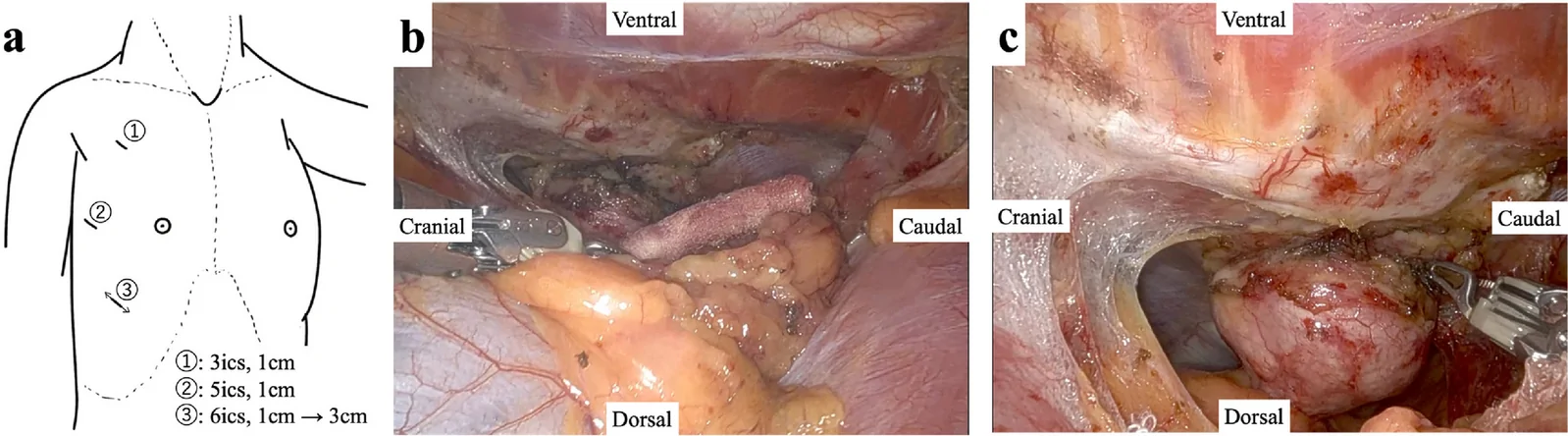
Surgical approach and intraoperative findings. **a** Schematic illustration of the three-port right-sided approach in the left semi-lateral decubitus position. The three ports were 1 cm incisions placed in the midaxillary line at the fifth intercostal space, the anterior axillary line at the third intercostal space, and anterior to the anterior axillary line at the sixth intercostal space. The sixth intercostal incision was subsequently extended to 3 cm for tumor extraction. **b** Intraoperative photograph showing the narrowed space between the heart and the sternum. **c** Intraoperative photograph showing the direct continuity of the tumor with the left sixth costal cartilage

The patient underwent RATS under general anesthesia with one-lung ventilation in the left semi-lateral decubitus position, using three 1-cm ports at the fifth intercostal space (midaxillary line), third intercostal space (anterior axillary line), and sixth intercostal space (anterior to the anterior axillary line) (Fig. 3a), with carbon dioxide insufflation at 5 mmHg. The tumor was not visible from the right thoracic cavity but was identified after incising the mediastinal pleura; dissection of its cranial aspect entered the left pleural cavity, where part of it was exposed, without adhesions. The tumor occupied the narrow space between the heart and sternum (Fig. 3b), contacting the left fifth through seventh costal cartilages with direct continuity to the sixth (Fig. 3c). Because the boundary between the tumor and normal cartilage could not be identified, the sixth costal cartilage was divided with bipolar energy applied through the robotic Maryland forceps, following the plane in which division was possible, and the specimen was removed in one piece; the sixth intercostal incision was extended to 3 cm for extraction. Operative time was 90 min, with 2 mL blood loss.

**Fig. 4:**
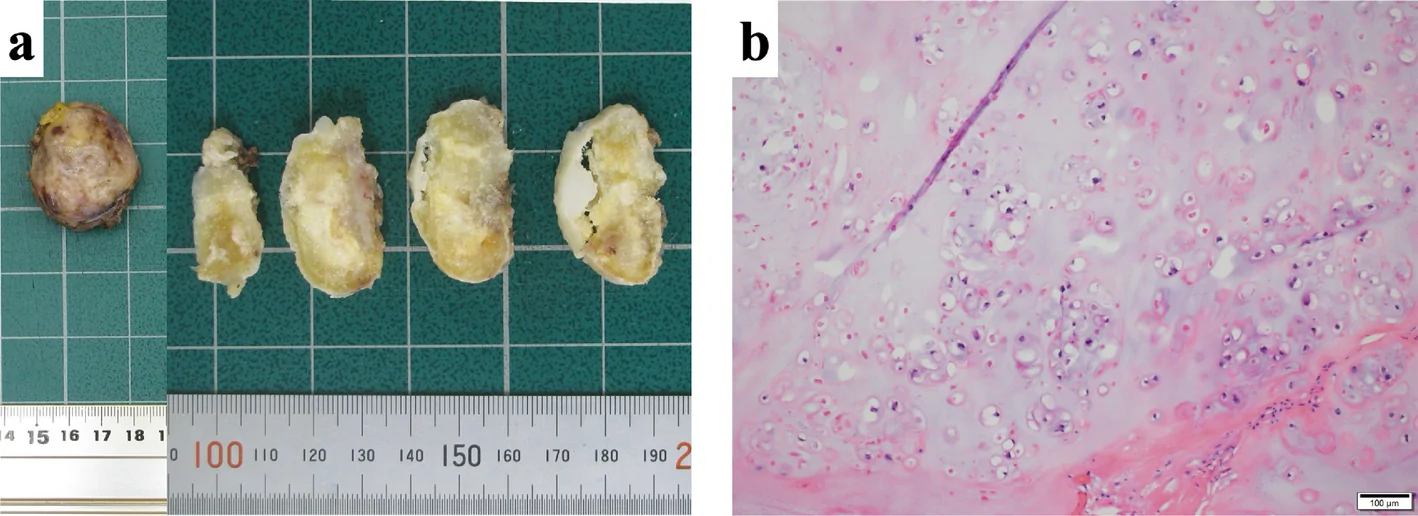
Histopathological findings of the resected specimen. **a** Gross examination of the resected specimen shows a well-circumscribed, firm mass with a glistening cartilaginous cut surface and focal calcification. **b** Hematoxylin and eosin staining reveals a proliferation of chondrocyte-like tumor cells that meet the histological criteria for grade 1 chondrosarcoma

The postoperative course was uneventful, and the patient was discharged on postoperative day 4. Gross examination showed a well-circumscribed, firm mass with a glistening cartilaginous surface and focal calcification (Fig. 4a). Hematoxylin and eosin staining showed chondrocyte-like tumor cells with mild nuclear atypia, consistent with grade 1 chondrosarcoma of the costal cartilage (Fig. 4b). No uninvolved costal cartilage was identified in the specimen and the tumor-cartilage boundary was indistinct; although no definite tumor was seen at the inked margin, sectioning and crush artifact precluded reliable assessment, so residual tumor could not be excluded (Rx) and the resection was at most marginal. CT and MRI one month after surgery showed no residual disease. Wide resection would ordinarily have been indicated, but surveillance was proposed given its morbidity and grade 1 histology; the patient agreed. No recurrence has been observed at 18 months.

## Discussion and conclusions

We encountered a case of costal cartilage chondrosarcoma continuous with the left sixth costal cartilage, in which the preoperative diagnosis was confidently, and incorrectly, a mature teratoma, and which was resected using a three-port right-sided RATS approach; to our knowledge, such a case has rarely been documented.

Mediastinal extension produced features overlapping with those of a primary anterior mediastinal neoplasm, particularly mature teratoma. Chondrosarcomas classically show ring-and-arc calcification on CT and marked T2 hyperintensity on MRI, reflecting the chondroid matrix. Calcification on CT is also a recognized feature of mature teratoma and did not raise suspicion. On retrospective review, bone-window and reformatted CT still did not demonstrate continuity with the costal cartilage (Fig. 1b-d). T1 hyperintensity was interpreted as fat, a hallmark of teratoma, supported by signal loss on fat-suppressed imaging (Fig. 2b). T2 imaging showed low signal within the calcified portion and high signal elsewhere (Fig. 2c); high T2 signal can reflect chondroid matrix but equally fluid or myxoid content within a teratoma, and was therefore non-specific. Well-defined borders, absence of invasion, low FDG uptake, and normal tumor markers collectively argued against malignancy. Several prior reports describe similar misdiagnoses: Østergaard et al. described a costal chondrosarcoma strongly suspected to be thymoma, in which low FDG uptake and absent calcification precluded a confident malignant diagnosis [2], and Yang et al. reported a case secondary to hereditary multiple exostoses requiring differentiation from teratoma, lymphoma, and chondroblastoma [6].

Given the tumor’s retrosternal location near the xiphoid process, median sternotomy was considered too invasive for a lesion presumed benign, and conventional VATS offered inadequate maneuverability in this confined corridor. We therefore selected a right-sided RATS approach, which provided adequate working space and unobstructed visualization, as reported for robotic chest wall resection in difficult regions [7, 8]. Intraoperative discovery of continuity between the tumor and the sixth costal cartilage raised the possibility of a chest wall rather than mediastinal origin. Conversion to a wider resection guided by frozen section was considered but not undertaken, and in retrospect this was a missed opportunity. Nosotti et al. obtained frozen section when a presumed thymoma proved adherent to the sternum and proceeded to en bloc sternal and costal cartilage resection [4]; conversely, Østergaard et al. reported frozen section of a costal chondrosarcoma read as benign, with grade 1 chondrosarcoma confirmed only on final pathology and a second operation with wider chest wall resection required thereafter [2]. Frozen section is therefore neither reliably conclusive nor dispensable, and preoperative suspicion remains the more dependable safeguard.

Re-resection would ordinarily have been favored. The board nevertheless judged surveillance reasonable, weighing the absence of residual disease on imaging, the grade 1 histology, and the extensive chest wall and diaphragmatic resection and reconstruction a wide margin would have required; surveillance comprises CT every three months, MRI every six months, and a low threshold for intervention. In a population-based series of 106 chest wall chondrosarcomas, Widhe and Bauer classified margins as wide (over 2 cm), marginal (tumor-free but under 2 cm), or intralesional, and reported local recurrence in 4% and 73% after wide and intralesional resection, with margin and grade as independent predictors [1]. Grade 1 outcomes in that series were not confined to wide margins, achieved in only 28%: local recurrence occurred in 2 of 19 grade 1 tumors, none metastasized, and 13 of 44 recurred after marginal versus 19 of 26 after intralesional resection [1]. The 4% figure reflects the margin obtained at the first operation; no data show re-resection restores it.

Local recurrence here is nonetheless lethal, nearly half of those recurring without metastases dying of local progression [1]; imaging cannot exclude microscopic residual disease, and wide resection remains standard. Our specimen was removed in one piece, but the cartilage was divided at the tumor interface rather than at a measured distance, so the resection was at best marginal and an intralesional margin cannot be excluded. No recurrence has been observed at 18 months, but the mean time to local recurrence in that series was 3.5 years, ranging to 14 years, with four recurrences beyond 10 years [1]. Our follow-up therefore falls far short of that interval. Costal cartilage origin rests on the intraoperative finding alone; neither imaging nor the specimen demonstrated the interface.

In conclusion, costal cartilage chondrosarcoma should be considered in the differential diagnosis of anterior mediastinal tumors, as it can closely mimic mature teratoma on imaging; preoperative recognition of this possibility is essential for appropriate oncological planning, since a minimally invasive approach must never compromise the surgical margin when malignancy is suspected. In this case, the right-sided RATS approach provided favorable visualization and maneuverability for a tumor in the confined retrosternal space, but its oncological safety cannot be generalized from a single case; RATS may be feasible in carefully selected patients in whom an adequate margin can be secured, warranting further validation in larger series.

## Data Availability

The datasets used in the current study are available from the corresponding author upon reasonable request.

## Abbreviations

AFP: Alpha-fetoprotein
CEA: Carcinoembryonic antigen
CT: Computed tomography
FDG: Fluorodeoxyglucose
MRI: Magnetic resonance imaging
PET-CT: Positron emission tomography-computed tomography
RATS: Robot-assisted thoracoscopic surgery
Rx: Residual tumor cannot be assessed
SCC: Squamous cell carcinoma antigen
VATS: Video-assisted thoracoscopic surgery
